# Asthma biomarkers in the age of biologics

**DOI:** 10.1186/s13223-017-0219-4

**Published:** 2017-11-17

**Authors:** Harold Kim, Anne K. Ellis, David Fischer, Mary Noseworthy, Ron Olivenstein, Kenneth R. Chapman, Jason Lee

**Affiliations:** 1 Division of Clinical Immunology & Allergy, Department of Medicine, Western University, 1151 Richmond St, London, ON N6A 5C1 Canada; 2 Division of Clinical Immunology & Allergy, Department of Medicine, McMaster University, 1280 Main Street West, Hamilton, ON L8S 4K1 Canada; 3 Division of Allergy & Immunology, Department of Medicine, Queen’s University, 15 Arch Street, Kingston, ON K7L 3N6 Canada; 4 Department of Biomedical and Molecular Sciences, School of Medicine, Queen’s University, Kingston, ON Canada; 5 Canadian Society of Allergy and Clinical Immunology, P.O. Box 51045, Orleans, ON K1E 3W4 Canada; 6 Alberta Children’s Hospital, University of Calgary, 2500 University Dr. NW, Calgary, AB T2N 1N4 Canada; 7 Division of Respiratory Medicine, Faculty of Medicine, McGill University, 3605 Rue De la Montagne, Montreal, QC H3G 2M1 Canada; 8 Acute Care Division, Montreal Chest Institute, 1001 Décarie Blvd, Montreal, QC H4A 3J1 Canada; 9 Asthma and Airway Centre, Toronto Western Hospital, University Health Network, 399 Bathurst Street, Toronto, ON M5T 2S8 Canada; 10 Division of Respirology, Department of Medicine, University of Toronto, 1 King’s College Circle, #3172, Toronto, ON M5S 1A8 Canada; 11 Toronto Allergy and Asthma Centre, 123 Edward St, Toronto, ON M5G 1E2 Canada; 12 Keenan Research Centre for Biomedical Science, St. Michael’s Hospital, 30 Bond St, Toronto, ON M5B 1W8 Canada; 13 Department of Surgery, School of Medicine, University of Toronto, 1 King’s College Circle, #3172, Toronto, ON M5S 1A8 Canada; 14 Evidence Based Medical Educator Inc., 123 Edward St., Suite 920, Toronto, ON M5G 1E2 Canada

**Keywords:** Asthma, Biomarkers, Phenotypes, Biologics

## Abstract

The heterogeneous nature of asthma has been understood for decades, but the precise categorization of asthma has taken on new clinical importance in the era of specific biologic therapy. The simple categories of allergic and non-allergic asthma have given way to more precise phenotypes that hint at underlying biologic mechanisms of variable airflow limitation and airways inflammation. Understanding these mechanisms is of particular importance for the approximately 10% of patients with severe asthma. Biomarkers that aid in phenotyping allow physicians to “personalize” treatment with targeted biologic agents. Unfortunately, testing for these biomarkers is not routine in patients whose asthma is refractory to standard therapy. Scientific advances in the recognition of sensitive and specific biomarkers are steadily outpacing the clinical availability of reliable and non-invasive assessment methods designed for the prompt and specific diagnosis, classification, treatment, and monitoring of severe asthma patients. This article provides a practical overview of current biomarkers and testing methods for prompt, effective management of patients with severe asthma that is refractory to standard therapy.

## Background

Asthma remains a significant worldwide health condition in terms of both prevalence and severity within all regions and amongst every age group. There are an estimated 235–334 million asthma sufferers worldwide [1, 2], Asthma is responsible for approximately 250,000 deaths annually [3]. In Canada, physician-diagnosed asthma was reported by approximately 2.4 million Canadians aged ≥ 12 years, corresponding to approximately 8.1% of the country’s population [4]. Asthma rates are higher in children; the National Longitudinal Survey of Children and Youth (from 1994/1995 to 2008/2009) determined an asthma prevalence of 9.8% in Canadian children aged 2–7 years [5].

The heterogeneous nature of asthma has been well established with the recognition of multiple pathways, mediators, and systems involved in triggering the characteristic airway inflammation and variable airflow limitation of asthma. Ongoing classification of different asthma phenotypes is a reflection of this heterogeneity. Indeed, severe asthma is often recognized as a specific asthma phenotype rather than an extreme manifestation of more commonplace asthma variants [6–8]. Severe asthma is defined by the joint European Respiratory Society/American Thoracic Society (ERS/ATS) guidelines according to the following criteria [9]:

- Requirement for treatment with high-dose inhaled corticosteroids (ICS) and a second controller (and/or systemic corticosteroids) to maintain control.
- Refractory to the treatment mentioned above.
- Incomplete management of comorbidities such as severe sinus disease or obesity.

The prevalence of severe, refractory asthma is generally estimated to be 5–10% of the total asthma population [9–14]. It is important to distinguish between asthma that is difficult to control and asthma that is truly severe. Initial assessment must rule out treatment confounders such as poor patient adherence and improper device technique as potential causes of suboptimal treatment response [9, 14–16]. The ERS/ATS guidelines cite reports indicating non-adherence as high as 32–56% [9]. Pooled data from 18 studies (January 1980 to October 2013) using electronic measurement of adherence to ICS among children with asthma determined a range of mean adherence rates of 28–71% (14 studies) and median adherence rates of 58–92% (4 studies) [17]. Although assessment of adherence can be challenging, physicians can encourage optimal self-management through open communication and shared decision making with the patient and family, education about the benefits of treatment and proper usage, and routine verification of medication usage and inhaler technique [15, 16]. Treatment regimens should take into consideration preferences that are important to the patient and caregiver [15].

Biologic agents have been shown to be effective and safe in patients with moderate to severe asthma but with variable response amongst patients with different phenotypes [18–30]. The emergence of specific and sensitive biomarkers has equipped the treating physician with important tools to tailor therapy towards optimal outcomes.

The objectives of this article are to:

- Evaluate the role of biomarkers in the identification of specific patient phenotypes towards selection of the most appropriate biologic for an individual patient.
- Describe testing options for various biomarkers with respect to their reliability, noninvasiveness, and accessibility.
- Review biologic agents established as safe and effective in the management of asthma resistant to standard treatment.

## Asthma phenotypes

From its origins in ancient Greek literature [31], the term “asthma” has evolved from a single disease entity, defined by a short list of clinical symptoms relating to the airway, to a broad term encompassing several distinct subgroups. The Merriam-Webster dictionary defines a phenotype as “the observable properties of an organism that are produced by the interactions of the genotype and the environment” [32].

The establishment of a definitive list of asthma phenotypes has been hindered by both the absence of a unified system of classification and by confounding comorbidities and co-existing conditions (Table 1) [33, 34]. Within this present uncertainty, several groups have attempted to identify and define the most prevalent phenotypes. The Asthma Phenotypes Task Force—a collaboration of the United States National Heart, Lung, and Blood Institute (NHLBI), the National Institute of Allergy and Infectious Diseases, the American Academy of Allergy, Asthma and Immunology, the ERS, and the ATS—proposed nine asthma phenotypes in three general categories (Table 2) [33]. Asthma phenotypes are generally separated according to allergy status, age of onset, and association with patient characteristics (e.g., exercise-induced, obesity related) [34–39].

**Table 1:** Comorbid conditions that complicate asthma phenotyping [33, 34]

Allergies Rhinosinusitis Gastroesophageal reflux disease Obstructive sleep apnea Smoking or exposure to second-hand smoke Obesity Hormonal influence Viruses and bacteria Occupational exposure Vocal cord dysfunction Food Osteopenia and osteoporosis Psychological problems (e.g., anxiety ± hyperventilation)	Churg-Strauss disease Pregnancy Chronic obstructive pulmonary disease Eczema Infections and vaccination Bronchiectasis and cystic fibrosis Exercise-induced bronchoconstriction Endocrine factors Conjunctivitis Congestive heart failure Pulmonary embolism Medications Primary ciliary dyskinesia

**Table 2:** Asthma phenotypes task force recommendations: asthma phenotypes [33]

Category	Phenotype
Trigger-induced asthma	(1) Allergic (2) Non-allergic (3) Aspirin-exacerbated respiratory disease (AERD) (4) Infection (5) Exercise-induced
Clinical presentation of asthma	(6) Pre-asthma wheezing in infants Episodic (viral) wheeze Multi-trigger wheezing (7) Exacerbation-prone asthma (8) Asthma associated with apparent irreversible airflow limitation
Inflammatory markers of asthma	(9) Eosinophilic and neutrophilic asthma

**Fig. 1:**
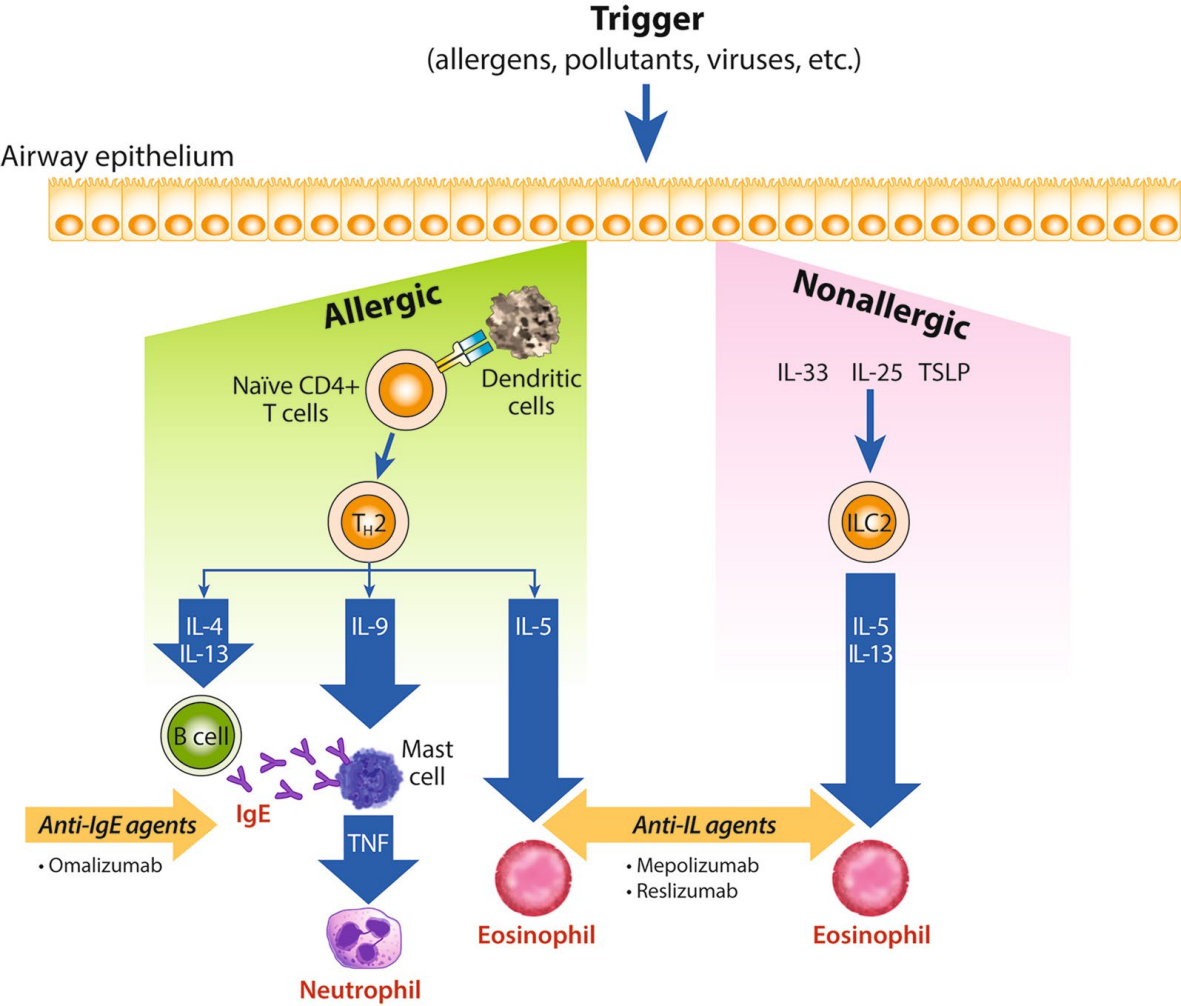
Allergic and non-allergic inflammatory asthma cascade. *IgE* immunoglobulin E, *IL* interleukin, *T*_*H*_ T helper, *TNF* tumour necrosis factor, *TSLP* thymic stromal lymphopoietin

Allergic asthma is widely identified as the most common phenotype [15, 33, 34, 40–43], particularly among children [44, 45]. Approximately 60% of asthma is considered allergic [41, 42]. Atopy was described in the NHLBI and National Asthma Education and Prevention Program (NAEPP) 2007 Expert Panel Report 3 as the “strongest identifiable predisposing factor” for the development of asthma [15]. Furthermore, multi-allergen screening to define atopy was cited as the sole core biomarker recommendation by an expert working group organized by National Institutes of Health institutes and federal agencies [46]. Inflammation in allergic asthma is initiated by the activity of antigen-presenting cells that promote the production of type 2 T helper (Th2) cells from naïve T lymphocytes. Th2 cells then mediate the allergic asthma pathway through proinflammatory cytokines—i.e., interleukins (IL)-4, IL-5, IL-9, and IL-13—leading to the production of immunoglobulin E (IgE) early in the cascade and, later, eosinophils (Fig. 1) [47]. Allergic asthma is typically identified based on sensitization, as determined by at least one positive skin prick test to a perennial and/or clinically relevant allergen or in vitro testing for IgE.

Non-allergic asthma has been shown to have one or more different pathways leading to airway inflammation. Cytokines originating in the epithelium (IL-25, IL-33, and thymic stromal lymphopoietin) activate type 2 innate lymphoid cells, from which IL-5 and IL-13 are produced and contribute to elevated eosinophil levels, mucus hypersecretion, and airway inflammation and hyperreactivity (Fig. 1) [47–49]. Non-allergic asthma tends to develop later in life and more predominantly in women than the allergic variety [33, 50, 51]. The prevalence of non-allergic asthma is generally considered to be 10–33% [51]. It appears to be associated with more severe asthma and a lower responsiveness to standard therapy [51]. Non-allergic asthma is diagnosed when allergic sensitization cannot be demonstrated using skin prick or in vitro IgE testing.

More precise identification of phenotypes may lead to the classification of asthma by endotypes. Endotypes are described as distinct asthma entities, as found in phenotype clusters, that are defined by a specific biological mechanism, providing a better understanding of the observable properties of that phenotype [34, 52].

## Biomarkers

The identification and continued refinement of asthma phenotypes has given rise to a more personalized, targeted management approach, particularly in patients with severe refractory asthma [53]. Biological markers aid in our understanding and recognition of phenotypes, help to identify other treatments most likely to be effective for the individual asthma patient with an inadequate response to first-line pharmacotherapy, and have the potential to assess treatment response. The ongoing Assessing Biomarkers in a Real-world Severe Asthma (ARIETTA) study is evaluating the relationship between asthma biomarkers and disease-related health outcomes in approximately 1200 patients with severe asthma within more than 20 countries [54]. Other collaborations such as the Unbiased Biomarkers for the Prediction of Respiratory Disease Outcomes (U-BIOPRED) project [55, 56] and the Airways Disease Endotyping for Personalized Therapeutics (ADEPT) study group [57] have also contributed findings on the utility of asthma biomarkers. Currently targeted asthma biomarkers are listed in Table 3.

**Table 3:** Currently used asthma biomarkers

Biomarker	Testing method	Phenotype	Role in allergic pathway	Associated cytokines	Associated biologic agents
IgE	Serum	Allergic (early onset)	Binds to FcεRI on mast cells, basophils, and antigen-presenting dendritic cells Activates the release of inflammatory mediators	IL-4, IL-13	Omalizumab
Eosinophil	Blood, sputum	Eosinophilic (late onset)—allergic and non-allergic	Modulates the immune response Promotes airway hyperresponsiveness and remodelling	IL-5	Mepolizumab, reslizumab, benralizumab
				IL-4, IL-13	Dupilumab
Neutrophil	Sputum	Neutrophilic	Significantly associated with severe asthma Accumulates in the airways Prominent in airway secretions during exacerbations	IL-8, IL-17	
Surrogate					
Periostin	Serum, sputum	Eosinophilic	Regulates eosinophil recruitment and eosinophilic tissue infiltration Active in Th2 mucosal inflammation, airway remodelling, and expression of inflammatory mediators	IL-4, IL-13	Lebrikizumab, tralokinumab, omalizumab
DPP-4	Serum	Eosinophilic, AERD	Stimulates the proliferation of bronchial smooth muscle cells and human fetal lung fibroblasts Promotes fibronectin production	IL-13	Tralokinumab

*DPP-4* dipeptidyl peptidase-4, *IgE* immunoglobulin E, *IL* interleukin

### IgE

Allergen-specific IgE is the predominant biomarker for allergic asthma [58–60]. Its production is stimulated early in the allergic asthma cascade by the release of IL-4, IL-5, and IL-13 through activated Th2 cells. IgE binds to FcεRI, which is expressed by several cells including mast cells, basophils, eosinophils, and B lymphocytes. The subsequent binding of allergens to allergen-specific IgE activates the release of proinflammatory mediators (e.g., tryptase, histamine, prostaglandins, leukotrienes), resulting in allergic symptoms [61–63]. Serum IgE levels have been shown to correlate closely with the presence and severity of asthma in adults, adolescents, and children [44, 58, 64–69]. It was also determined that serum IgE levels were associated with airway hyper-responsiveness, even in patients without a history of asthma symptoms or atopy [70].

### Eosinophils

Produced in the bone marrow, eosinophils are recruited into areas of inflammation through complex interactions among cytokines and several other molecules [71]. Eosinophils depend particularly on IL-5 for their maturation, activation, and survival [47, 48]. Eosinophils can be measured in both blood and sputum; the values differ significantly as a reflection of the local (sputum) versus systemic (blood) nature of these measurement methods (*see the sections on blood and sputum testing*). As with IgE, the presence of significant eosinophilia is associated with severe asthma; [8, 72–76] however, severe asthma is not identified exclusively with eosinophilia [6]. Kamath et al. argued against eosinophil being the most important effector cells in the pathogenesis of asthma based on findings that demonstrated: (1) the presence of airway lumen eosinophilic inflammation is present in only 50% of patients; (2) intense eosinophilic inflammation alone does not induce asthma; and (3) a high frequency of exacerbations occurs even in the absence of airway eosinophilia [77]. Thus, the specific role of eosinophils in asthma severity remains a point of controversy [78].

### Neutrophils

Neutrophils are the most abundant cell type found in induced sputum samples, independent of asthma status [76, 79]. Markedly increased numbers of airway neutrophils were found in association with severe asthma and in patients experiencing acute severe exacerbations [76, 79–82]. Neutrophils are triggered by IL-8 and produce enzymes and other factors that contribute to eosinophil activity [83]. Although it has been asserted that eosinophilic and neutrophilic asthma are not mutually exclusive conditions [77], neutrophilic inflammation has been shown to be associated with lower levels of forced expiratory volume in 1 s (FEV_1_), particularly post bronchodilator use, and methacholine responsiveness independent of eosinophilia [76, 80].

### Cytokines

As indicated in the descriptions of the previously listed biomarkers, both the allergic and non-allergic pathways of asthma inflammation are mediated at multiple points by the various ILs. IL-4, IL-5, and IL-13 originate from Th2 lymphocytes and mast cells, and are involved in the mediation of Th2 inflammation [13, 84–88]. IL-5 selectively acts on eosinophils and basophils; it promotes eosinophil recruitment, differentiation, maturation, and activation [84, 89, 90], as well as the maturation, growth, activation, and survival of basophils [91]. IL-4 and IL-13 are active in IgE synthesis, and mediate eosinophil recruitment and activation, mucus secretion, and airway remodelling [84, 92, 93]. IL-13 is also involved in the activation and proliferation of bronchial fibroblasts that increase in bronchial hyper-responsiveness [94, 95].

IL-9 and IL-11 have been shown to be more specifically involved in the severe asthma cascade [13, 96]. IL-9 is derived from Th2 cells, but can also be produced by a host of other cells under specific conditions [97]. It is a significant contributor to the differentiation and proliferation of mast cells [98, 99]. IL-11 has been shown to be involved in chronic airway remodelling [96]. IL-17 is another cytokine that has been correlated with severe asthma [100–102]. Researchers have found an association between IL-17 and increased neutrophilic airway inflammation [103], as well as with the induction of IL-6 and the production of IL-8 from fibroblasts [104].

Cytokines such as IL-25 and IL-33 are active in the early stages of the inflammatory cascades of both allergic and non-allergic asthma. IL-25 and IL-33 are involved in the stimulation of type 2 innate lymphoid cells (ILC2s), which are another source of IL-5, IL-13, and (to a lesser extent) IL-4 [47, 105–107]. A number of genome-wide association studies have identified IL-33 and its receptor genes as highly implicated in the development of asthma [108].

IL-22 appears to exhibit both pro- and anti-inflammatory activity. It is found in high concentration in patients with severe asthma [109], and blockage of IL-22 significantly reduces eosinophilic inflammation, eosinophil recruitment, mucus production, and Th2 cytokine production in an asthmatic mouse model [110]. In the same study, however, IL-22 inhibition resulted in increased Th2 cytokine production and greater allergic lung inflammation. This effect may be secondary to the inhibition of IL-22, with the increased production of IL-25 in lung epithelial cells [111].

IL-23 is involved in the differentiation of Th17 cells and in the production of IL-17 and IL-22 [112–115]. It is also a key regulator of IL-17 [116].

### Periostin

The extracellular matrix protein, periostin, has been found to be a downstream molecule of IL-4 and IL-13, which upregulate periostin expression in bronchial epithelial cells and fibroblasts [117–119]. IL-13-stimulated epithelial cells secrete periostin into the extracellular matrix. It plays a role in the regulation of eosinophil recruitment and tissue infiltration, accumulation in Th2 mucosal inflammation, and is also involved in airway remodelling and increased expression of inflammatory mediators [120, 121]. Periostin has been proposed as a surrogate biomarker for type 2 immunity to predict the efficacy of treatments targeting IL-13 and IgE [122].

### Dipeptidyl peptidase 4 (DPP-4)

Little research has been published on the activity of DPP-4 in human asthma. Rat models have shown increased enzyme activity further to allergen challenge [123], and topical (but not oral) DPP-4 inhibition in rats reduced airway hyper-responsiveness [124]. In human subjects, IL-13 was identified as a significant inducer of DPP-4 [125]. DPP-4 has been found to be a stimulator of proliferation of bronchial smooth muscle cells and human fetal lung fibroblasts, and it promotes the production of fibronectin [126]. There is evidence that DPP-4 may serve as a biomarker for aspirin-exacerbated respiratory disease [127].

## Currently available tests: practical points for optimal use

The identification of biomarkers has been shown to provide valuable information and guidance for selecting therapies that can result in best patient outcomes. However, in order for this information to become actionable, the testing method must be sufficiently sensitive and specific. The practicality of a biomarker test is inversely proportional to its invasiveness. Also, availability and cost to the healthcare system and/or the patient are also necessary to consider for the selection of the most appropriate test.

The current diagnosis of asthma through a combination of clinical history with pulmonary function testing and methacholine or exercise challenge test [128] does not specifically characterize or quantify airway inflammation. Bronchoscopy/biopsy and bronchoalveolar lavage continue to be useful for the assessment of the asthma patient and for detection of asthma biomarkers, and are safe even in severe asthma when proper precautions are used [129–135]. However, the invasive nature of these procedures limits their usefulness, particularly for the purpose of ongoing monitoring.

As a standard approach, all patients with moderate to severe or difficult-to-treat asthma should undergo the following tests:

- Aeroallergen skin prick testing.
- Total IgE.
- Complete blood count (CBC) with differential, including blood eosinophil level.

These tests are reliable and are easily accessed in the Canadian healthcare system. Additional tests may be considered, depending on the specific patient profile. Details of available tests are presented below.

### Allergy skin testing

Skin prick testing is a widely available, inexpensive, simple, and minimally invasive method to assess the patient’s allergic status to an IgE-mediated allergen [16, 136]. Another advantage is that results are generally known within 15–20 min of application of the reagents to the skin. Skin prick testing can also be used to test for atopic response to less common allergens for which no specific IgE antibody test is available, such as some medications [136]. The test has been found to be sensitive when performed by an experienced tester with standardized extracts, and reproducible [16, 136, 137]. In this population, technique is as important as testing device to maximize accuracy of outcome [138]. The need for a consensus on minimal tester standards has been highlighted to minimize the gap between expert recommendation and daily practice [139].

### Blood IgE testing

Testing of a patient’s blood remains an important component in the diagnosis of asthma; the detection of elevated IgE levels and eosinophils can be used to assist in identifying allergic sensitivity. Specific IgE (i.e., IgE directed against a specific allergen) and eosinophil count were confirmed as the most consistent biomarkers to measure the risk of asthma in children [140]. Agreement between in vitro specific IgE and skin prick testing was 85–90%, depending on the allergen and testing method [136]. In a comparison between the two methods to detect airway reactivity to house dust mite, skin prick testing was more sensitive but IgE testing was more specific [141]. Measurement of specific and total (i.e., sum of all specific IgE levels) serum IgE levels can be useful in the diagnosis of asthma and to distinguish between allergic and non-allergic asthma [61, 62, 68, 142–144]. Measurement of total IgE is also essential to determine suitable candidates for treatment with omalizumab (i.e., those with serum total IgE levels in the range of 30–700 IU/mL), as well as to establish proper dosing. Specific IgE does not improve reliability over skin prick testing and is more expensive; however, it can be advantageous to use in uncooperative patients, those who have extensive skin conditions, or if their allergy history indicated a risk of anaphylaxis [135]. If specific IgE is being ordered for consideration of selecting an appropriate biologic agent, screening for perennial allergens such as dust mite would have the best rationale. The mean total serum IgE concentration for healthy adults was identified in 1969 as 250 ng/mL, compared with a mean level of up to 2800 ng/mL in atopic individuals and 1600 ng/mL for those with extrinsic asthma [145]. Normal total serum IgE is now understood to be age-dependent [145, 146]. The reference intervals range from 2 to 34 IU/mL in infants aged 6–12 months to 2–696 IU/mL in children aged 9–12 years, and then decreases to 2–214 IU/mL for adults aged ≥ 18 years (Table 4) [147]. Interestingly, total and specific serum IgE were found to decrease with age in patients with asthma [148].

**Table 4:** Total serum IgE reference intervals. Reproduced from [147]

Age	Reference interval (IU/mL)
6–12 months	2–34
1–2 years	2–97
3 years	2–199
4–6 years	2–307
7–8 years	2–403
9–12 years	2–696
13–15 years	2–629
16–17 years	2–537
≥ 18 years	2–214

### Other blood tests

Blood eosinophil count is an accurate diagnostic indicator of mild, moderate, and severe eosinophilic asthma [149–153]. Blood eosinophil count and the level of serum eosinophil protein were shown to be indicators of the short-term increases in asthma symptoms (wheezing, cough, dyspnea, and exercise-induced asthma) and bronchial hyper-responsiveness, reduction in FEV_1_, and the need for corticosteroid treatment in patients with mild to moderate asthma [154]. Clinical trials involving mepolizumab found that the rate of clinically significant asthma exacerbations varied according to blood eosinophil level, as opposed to sputum [26, 150, 151]. The normal range of blood eosinophil count is 30–350 cells/µL; [155] however, there is controversy with respect to the cut off level associated with increased risk of asthma complications. Mepolizumab trials employed blood eosinophil cut offs of ≥ 150 to ≥ 300 cells/µL [24–26, 151, 156]. This is in line with the findings of the Epidemiological study on the Genetics and Environment of Asthma group, who concluded that a blood eosinophil level ≥ 250 cells/µL correlated with more active asthma (i.e., lower FEV_1_) [157]. However, several studies determined that poor asthma control was associated with a higher eosinophil cut off. A large-scale (N = 130,248) UK cohort study used negative binomial regression to identify that poorer asthma control and more severe exacerbations were experienced by patients with blood eosinophil counts > 400 cells/µL [158]. Zieger et al. likewise concluded that a blood eosinophil count > 400 cells/µL was an independent risk factor for asthma exacerbations and asthma-related emergency department visits or hospitalizations [153].

### Other useful evaluations

#### Serum periostin

Elevated serum levels of periostin have been associated with asthma activity and severity, and with the presence of late-onset eosinophilic asthma [159, 160]. Organizers of the Bronchoscopic Exploratory Research Study of Biomarkers in Corticosteroid-refractory Asthma (BOBCAT) concluded that serum periostin is potentially useful for the selection of agents that target Th2 inflammation [161]. In their study, eosinophil-high and eosinophil-low subjects were differentiated by periostin with a positive predictive value of 93%, and serum periostin levels were more consistent than blood eosinophil counts. This group also determined that serum periostin was a significantly better predictor of airway eosinophilia than other biomarkers tested, including IgE, peripheral blood eosinophils, fractional exhaled nitric oxide (FeNO), and YKL-40 [161]. Wagener et al. concluded, however, that periostin did not distinguish between eosinophilic and non-eosinophilic airway inflammation [149]. A precise definition of high periostin levels has not been established; most studies involving lebrikizumab employed the median periostin level across the study cohort as the cut off between high- and low-periostin groups [162–164]. The requirement of an enzyme-linked immunosorbent assay limits the availability of this test, and periostin levels are rarely obtained outside of clinical research.

#### Induced sputum

Induction of a sputum sample is an effective and non-invasive method of biomarker collection in asthma patients as young as 6 years of age [128, 165, 166]. This test produces a differential count of 400 inflammatory cell types, including eosinophils, neutrophils, macrophages, lymphocytes, and epithelial cells. Reproducibility, validity, and responsiveness have been demonstrated [167, 168]. In a population of healthy adults in Western Canada, Davidson et al. determined the following mean (± standard deviation) differential cell percentages: neutrophils 50.3 ± 23.5%; eosinophils 1.4 ± 2.3%; macrophages 43 ± 22.8%; lymphocytes 2.6 ± 5.2%; and bronchial epithelial cells 2.2 ± 4.8% [169]. Induced sputum has also been instrumental in identifying four inflammatory phenotypes: eosinophilic, neutrophilic, paucigranulocytic (i.e., normal neutrophil and eosinophil levels), and mixed granulocytic (i.e., elevated levels of both neutrophils and eosinophils) [166]. The disadvantages of induced sputum counts in Canada include high cost, technical demand, required time, and limited availability to only a few sites across Canada.

The Canadian Thoracic Society’s (CTS) 2012 asthma guidelines identified a mean differential sputum eosinophil count of < 2–3% as normal [128]. An elevated eosinophil count is associated with symptomatic asthma. During a response to airborne allergen exposure [167], ICs were shown to reduce eosinophil count [167, 170, 171], and a systematic review by Petsky et al. found that asthma treatment adjusted to sputum eosinophil count was associated with a significant reduction in the number of exacerbations [172]. The CTS guidelines suggest that sputum eosinophil counts be measured in adult asthma patients for the adjustment of anti-inflammatory treatment [128], and conducted in conjunction with standard asthma control assessment.

Sputum periostin is associated with persistent airflow limitation, as well as ICS resistance in eosinophilic asthma [173]. It is also a potential marker for airway remodelling [174]. Simpson et al. determined that periostin levels are significantly lower in sputum than in serum, and while both sputum and serum periostin levels are significantly related to sputum eosinophil levels, neither exhibits a high level of prediction of the presence of eosinophilic asthma [175].

#### FeNO

The generation of nitric oxide in the airways is indicative of Th2 inflammation [176, 177]. Study results conflict regarding the ability of FeNO to classify asthma severity [178–181]. In a six-year longitudinal study of patients with difficult-to-treat asthma, van Veen et al. determined that FeNO can predict accelerated decline of lung function [182]. FeNO assessed airway inflammation as accurately as induced sputum analysis [183], and predicted asthma relapse in asymptomatic children in the month after ICS discontinuation [184].

The CTS guidelines have not recommended routine use of FeNO for the adjustment of ICS dose, citing insufficient evidence [128]. This conclusion is in line with guidelines published by the ERS/ATS, the NHLBI/NAEPP, and the British Thoracic Society/Scottish Intercollegiate Guidelines Network [9, 15, 185]. However, FeNO is supported by the ATS for the detection of eosinophilic airway inflammation, assessing the potential need for and probability of response to ICS, and evaluating ICS adherence [186]. FeNO is simple to perform, usable in infants and preschool children, and results are immediately available; however, its relative sensitivity and specificity for eosinophilic inflammation are uncertain and accurate FeNO measurement is confounded by atopic status, smoking, and ICS use. It is available in most asthma clinics and some specialist clinics.

## Biomarker-guided management options

### Available biologics

The advent of biologic agents has revolutionized the management of patients with severe refractory asthma. These agents target different components of the inflammatory cascade, and are indicated for specific patient phenotypes (Table 5). Their current or anticipated availability suggests a provisional role for the use of biomarkers in the selection of biologics for severe asthma therapy.

**Table 5:** Approved and investigational biologic agents

Approved agent	Indication	Therapeutic target	Biomarkers	Dosing	
Omalizumab (Xolair^®^)	Moderate to severe persistent allergic asthma Positive skin test or in vitro reactivity to a perennial aeroallergen Patient is inadequately controlled with ICS ≥6 years of age; add-on therapy for 6–11 years of age Chronic idiopathic urticaria Symptomatic despite H1 antihistamine treatment ≥12 years of age US As Canadian PM, except that eligible patient age is ≥ 6 years EU Add-on therapy to improve control of severe persistent allergic (convincing IgE-mediated) asthma in patients aged ≥ 6 years Positive skin test or in vitro reactivity to a perennial aeroallergen + frequent daytime symptoms or night-time awakenings ≥12 years: reduced lung function (FEV_1_ < 80%) Multiple documented exacerbations despite daily high-dose ICS + long-acting inhaled beta_2_-agonist	IgE	IgE (serum) Periostin (serum, sputum)	75–375 mg SC every 2–4 weeks Dose determined by serum total IgE level and body weight	
Mepolizumab (Nucala^®^)	Severe eosinophilic asthma Add-on maintenance treatment ≥ 12 years of age Patient is inadequately controlled with high-dose ICS and ≥ 1 additional asthma controller Blood eosinophil count ≥ 150 cells/μL at initiation of treatment or ≥ 300 cells/μL in the past 12 months US As Canadian PM No details provided on lack of control on other asthma medication or specific blood eosinophil level EU As Canadian PM Specifies refractory nature of severe eosinophilic asthma Adult patients No details provided on specific blood eosinophil level	IL-5	Eosinophil (blood, sputum)	100 mg SC every 4 weeks	
Reslizumab (Cinqair™)	Severe eosinophilic asthma Add-on maintenance treatment ≥ 18 years of age Patient is inadequately controlled with medium- to high-dose ICS and ≥ 1 additional asthma controller Blood eosinophil count ≥ 400 cells/μL at initiation of treatment US As Canadian PM No details provided on lack of control on other asthma medication or specific blood eosinophil level EU As Canadian PM No details provided on specific blood eosinophil level Specifies high-dose ICS	IL-5	Eosinophil (blood, sputum)	3 mg/kg IV (20–50 min) every 4 weeks	

Investigational agent	Therapeutic target	Biomarkers	Study population(s)	Study dosing	Study results
Benralizumab	IL-5Rα	Eosinophil (blood, sputum)	Patients with severe eosinophilic asthma (blood eosinophil count ≥ 300 cells/µL) ≥ 12 years of age Uncontrolled (≥ 2 exacerbations) despite high-dose ICS and LABA use	30 mg SC every 4 or 8 weeks	Significant reduction of annual asthma exacerbation rate Significantly improved prebronchodilator FEV_1_
Dupilumab	IL-4/IL-13	Eosinophil (blood, sputum)	Patients with uncontrolled persistent asthma ≥ 18 years of age Taking medium- to high-dose ICS and a LABA	200 or 300 mg SC every 2 weeks or every 4 weeks	Significant increases in FEV_1_ Reduction in severe exacerbations
Lebrikizumab	IL-13	Periostin Eosinophil (blood)	Patients with uncontrolled asthma ≥ 18 years of age Pre-bronchodilator FEV_1_ 40–80% predicted Periostin ≥ 50 ng/mL or blood eosinophils ≥ 300 cells/μL ICS and ≥ 1 controller medication	37.5 or 125 mg SC every 4 weeks	No consistent significant reduction in exacerbations
Tralokinumab	IL-13	Periostin DPP-4	Patients with severe uncontrolled asthma ≥ 18 years of age Taking high-dose ICS and a LABA	300 mg SC every 2 or 4 weeks	No significant reduction in exacerbation rate Dosing every 2 weeks significantly improved prebronchodilator FEV_1_

*FEV*_*1*_ forced expiratory volume in 1 s, *LABA* long-acting beta2 agonist

#### Omalizumab (anti-IgE)

Omalizumab was approved in 2005 by Health Canada, and is indicated for the management of adult and pediatric (aged ≥ 6 years) patients with moderate to severe persistent asthma that is uncontrolled by ICS, and who exhibit allergic reactivity to a clinically relevant aeroallergen [187]. Initial trials demonstrating the safety and effectiveness of omalizumab in the management of moderate to severe allergic asthma [18–23] are supported by long-term (≥ 52 weeks) and real-world studies [188–191] and more than 400,000 patient-years of experience [192]. It is recommended as Step 5 add-on treatment in the 2016 Global Initiative for Asthma (GINA) guidelines for patients with moderate to severe allergic asthma [16]. Omalizumab binds IgE with high affinity and competitively inhibits its interaction with FcεRI, leading to the reduced expression of FcεRI on mast cells, basophils, and dendritic cells [60, 187]. The presence of omalizumab:IgE complexes increases serum total IgE levels after the agent has been initiated and these levels may remain elevated for up to 1 year after omalizumab has been discontinued. Thus, caution is indicated against basing reassessment of the dosing regimen on serum total IgE levels taken during this time period [187]. Although IgE is the principal biomarker when considering the administration of omalizumab in treatment-refractory asthma patients, other biomarkers may be valuable in guiding omalizumab use. Investigators of the EXTRA study noted that reductions in exacerbations associated with omalizumab use versus placebo were substantially greater in high- versus low-biomarker subgroups for all biomarkers studied: eosinophils, periostin, and FeNO [176]. Serum IgE and periostin levels were determined to be useful markers of response to omalizumab [193].

#### Mepolizumab (IL-5 inhibitor)

Mepolizumab was approved by Health Canada in 2015 for the add-on maintenance treatment of adult patients aged ≥ 18 years with severe eosinophilic asthma that is uncontrolled with ICS and an additional asthma agent [194]. Eosinophilic asthma was defined in mepolizumab trials as a blood eosinophil count of ≥ 150 cells/μL at initiation of mepolizumab or ≥ 300 cells/μL in the previous 12 months. Mepolizumab binds to IL-5 with high affinity, disrupting the production and survival of eosinophils. The agent was proven safe and effective in three randomized, double-blind clinical trials [24–26], and benefit beyond 48 months was also determined [195, 196]. The 2016 GINA guidelines added mepolizumab to its recommended Step 5 treatment options for patients aged ≥ 12 years with severe eosinophilic asthma [16]. Blood eosinophil count was found to be a more reliable marker of mepolizumab activity than sputum eosinophil measurement [26, 151]. Early studies that failed to demonstrate clinical improvement (airway hyperreactivity, peak expiratory flow, and FEV_1_) despite marked reductions in blood and sputum eosinophil count underline the importance of biomarker use for identification of appropriate candidates.

#### Reslizumab (IL-5 inhibitor)

Reslizumab was approved by Health Canada in 2016. Like mepolizumab, reslizumab is indicated for add-on maintenance treatment of patients aged ≥ 18 years with severe eosinophilic asthma—defined in reslizumab trials as a blood eosinophil count ≥ 400 cells/µL—and who are inadequately controlled with ICS and an additional asthma medication [197]. Reslizumab received its approval based on two randomized, double-blind trials [29]. Although blood eosinophil count is the approved measure for determination of eligibility to take reslizumab, this agent has also shown the ability to reduce sputum eosinophil levels [28].

### Investigational biologics

Several biologics are currently being tested in Phase III trials to confirm their safety and efficacy in asthma patients.

Benralizumab binds to the α subunit of the IL-5 receptor and reduces the number of IL-5Rα-producing cells through its antibody-directed, cell-mediated cytotoxic effect on eosinophils and basophils [198]. In two Phase III trials, benralizumab significantly reduced asthma exacerbations and improved pre-bronchodilator FEV_1_ [199, 200]. Patient stratification in these two trials was by baseline blood eosinophil count (≥ 300 cells/μL versus < 300 cells/μL).

Dupilumab acts on the α subunit of the IL-4 receptor and blocks signal transduction of both IL-4 and IL-13 [201]. It produced a greater reduction of exacerbations in patients with persistent moderate-to-severe asthma and eosinophilia compared with placebo [202]. Eligible patients had elevated eosinophil counts according to blood or sputum screening; in this study by Wenzel et al., the specific cut-off levels were ≥ 300 cells/μL for blood and ≥ 3% for sputum eosinophil. In a Phase IIb trial, dupilumab was associated with improvements in lung function and severe exacerbations in patients with uncontrolled persistent asthma regardless of baseline eosinophil count [203].

IL-13 inhibition is the target of two other experimental agents, lebrikizumab and tralokinumab [204, 205]. In parallel Phase III trials, lebrikizumab appeared to reduce IL-13, but asthma exacerbations were not significantly reduced [206]. Further lebrikizumab trials have been suspended. Tralokinumab failed to meet its primary endpoint of a significant reduction in the rate of asthma exacerbations over 52 weeks in the Phase III STRATOS 1 trial [207]. The ongoing STRATOS 2 trial is analyzing this same primary outcome in a subset of patients identified with periostin and DPP-4 measurement as having high IL-13 activity.

## Conclusion

It has been established that asthma is a heterogeneous condition, comprising a phenotypic spectrum of patient populations. Under this broad term, severe asthma itself covers a series of subgroups with specific characteristics, symptom profiles, and biochemical mechanisms of disease. Biologic agents represent a significant opportunity to administer individualized treatment for patients who do not respond to traditional asthma therapy. In this review, we have shown the importance of biomarkers to identify which patient phenotypes can be expected to derive the greatest benefit from these agents, and, for some, as indicators of treatment response. All patients with asthma in whom initiation of biologic therapy is being considered should undergo aeroallergen skin prick testing and IgE measurement to assess for the allergic asthma phenotype, and a CBC with differential to assess for elevated eosinophil levels. These biomarkers are both readily accessible and useful to provide accurate clinical information about the underlying asthma phenotype. The optimization of biomarker testing methods by combining greatest sensitivity and specificity with non-invasiveness, availability, and affordability is critical to the continued advancement of asthma control.
